# Supplementary material for: Effect of Biogenic Amine-Degrading Lactobacillus on the Biogenic Amines and Quality in Fermented Lamb Jerky
Source: Foods. 2022 Jul 12;11(14):2057. doi: 10.3390/foods11142057 (PMC9322946; doi:10.3390/foods11142057)
Supplement: Supplementary file 1 [file foods-11-02057-s001.zip › foods-1772390-supplementary.pdf]

foods

Table S1. Electronic nose response values in fermented lamb jerky during ripening process.

	Stage	CO	PL-4	BL4-8	CL4-3	X3-2B
W1C	Salting	1.22±0^A	1.22±0^A	1.21±0^A	1.22±0^A	1.21±0^A
	Fermentation	1.21±0^B	1.2±0^B	1.2±0^B	1.14±0^D	1.18±0^B
	Low-temperature roasting	1.13±0^D	1.14±0^D	1.15±0^D	1.16±0^C	1.17±0^C
	High-temperature roasting	1.16±0^C	1.16±0^C	1.16±0^C	1.17±0^B	1.17±0^C
W5S	Salting	1.13±0^D	1.14±0^D	1.14±0^D	1.14±0^D	1.16±0^C
	Fermentation	1.14±0^C	1.16±0^B	1.16±0^B	1.15±0^C	1.17±0^B
	Low-temperature roasting	1.15±0^B	1.17±0^A	1.17±0^A	1.16±0^A	1.18±0^A
	High-temperature roasting	1.13±0^A	1.14±0^C	1.14±0^C	1.14±0^B	1.16±0^D
W3C	Salting	1.1±0^A	1.1±0^A	1.1±0^A	1.1±0^A	1.09±0^A
	Fermentation	1.09±0^B	1.09±0^B	1.09±0^A	1.08±0^B	1.08±0^C
	Low-temperature roasting	1.08±0^C	1.07±0^D	1.07±0^B	1.08±0^C	1.08±0^B
	High-temperature roasting	1.07±0^D	1.07±0^C	1.07±0^B	1.07±0^D	1.08±0^C
W6S	Salting	1.01±0^C	1.01±0^C	1.03±0^C	1.02±0^C	1.04±0^C
	Fermentation	0.99±0^D	1.02±0^B	1.04±0^B	1.03±0^A	1.16±0.01^A
	Low-temperature roasting	1.03±0^A	1.03±0^A	1.02±0^D	1.02±0^B	1.01±0^D
	High-temperature roasting	1.02±0^B	1.01±0^D	1.06±0^A	1.01±0^D	1.05±0^B
W5C	Salting	1.14±0^A	1.13±0^A	1.13±0^A	1.13±0^A	1.13±0^A
	Fermentation	1.13±0^B	1.13±0^B	1.13±0^B	1.12±0^D	1.12±0^C
	Low-temperature roasting	1.12±0^D	1.12±0^C	1.12±0^C	1.12±0^C	1.13±0^B
	High-temperature roasting	1.13±0^C	1.13±0^B	1.12±0^B	1.13±0^B	1.12±0^B
W1S	Salting	1.2±0^D	1.29±0^D	1.35±0^C	1.26±0^D	1.38±0^D
	Fermentation	1.3±0^C	1.34±0^C	1.27±0^D	1.4±0^C	1.45±0.01^C
	Low-temperature roasting	1.43±0^B	1.73±0^A	1.58±0^B	1.47±0^B	1.48±0^B
	High-temperature roasting	1.74±0^A	1.58±0^B	1.64±0^A	1.61±0^A	1.57±0^A
W1W	Salting	1.11±0^D	1.13±0^D	1.13±0^D	1.15±0^C	1.16±0^D
	Fermentation	1.22±0^B	1.22±0^C	1.16±0^C	1.08±0^D	1.2±0^C
	Low-temperature roasting	1.13±0^C	1.39±0^B	1.42±0^B	1.26±0^B	1.34±0^B
	High-temperature roasting	1.59±0^A	1.54±0^A	1.52±0^A	1.56±0^A	1.49±0^A
W2S	Salting	1.04±0^D	1.06±0^D	1.08±0^D	1.06±0^D	1.08±0^C
	Fermentation	1.07±0^C	1.08±0^C	1.09±0^C	1.2±0^A	1.12±0^B
	Low-temperature roasting	1.21±0^A	1.24±0^A	1.17±0^A	1.15±0^B	1.15±0^A
	High-temperature roasting	1.18±0^B	1.16±0^B	1.17±0^B	1.15±0^C	1.15±0^A
W2W	Salting	1.09±0^C	1.09±0^D	1.09±0^D	1.1±0^B	1.1±0^C
	Fermentation	1.11±0^B	1.11±0^B	1.09±0^C	1±0^C	1.1±0^C
	Low-temperature roasting	1.02±0^D	1.1±0^C	1.16±0^B	1.1±0^B	1.15±0^B
	High-temperature roasting	1.19±0^A	1.17±0^A	1.18±0^A	1.19±0^A	1.17±0^A
W3S	Salting	1.02±0^B	1.02±0^D	1.05±0^A	1.02±0^C	1.02±0^B

Fermentation	1.01±0[D]	1.03±0[B]	1.04±0[B]	1.17±0[A]	1.05±0[A]
Low-temperature roasting	1.17±0[A]	1.11±0[A]	1.04±0[A]	1.07±0.01[B]	1.05±0[A]
High-temperature roasting	1.02±0[C]	1.02±0[C]	1.02±0[C]	1±0[D]	1.01±0[C]

[A,B,C,D]: Mean values followed different uppercase letter in the same column indicate significant difference ($p<0.05$). CO: control without starter culture; PL-4: with PL-4 *Lactobacillus plantarum*; BL4-8: with BL4-8 *Lactobacillus plantarum*; CL4-3: with CL4-3 *Lactobacillus plantarum*; X3-2B: with X3-2B *Lactobacillus plantarum*.
